# Tonically Active α_5_GABA_A_ Receptors Reduce Motoneuron Excitability and Decrease the Monosynaptic Reflex

**DOI:** 10.3389/fncel.2017.00283

**Published:** 2017-09-19

**Authors:** Martha Canto-Bustos, Emanuel Loeza-Alcocer, Carlos A. Cuellar, Paulina Osuna, David Elias-Viñas, Vinicio Granados-Soto, Elías Manjarrez, Ricardo Felix, Rodolfo Delgado-Lezama

**Affiliations:** ^1^Department of Neuroscience, Center for the Neural Basis of Cognition, University of Pittsburgh Pittsburgh, PA, United States; ^2^Department of Neurobiology, University of Pittsburgh School of Medicine Pittsburgh, PA, United States; ^3^Laboratory of Neuronal Engineering, Mayo Clinic Minnesota Rochester, MN, United States; ^4^Departamento de Fisiología, Biofísica y Neurociencias, Cinvestav Mexico City, Mexico; ^5^Departamento de Ingeniería Electrica, Cinvestav Mexico City, Mexico; ^6^Departamento de Farmacobiología, Cinvestav Mexico City, Mexico; ^7^Instituto de Fisiología, Benemérita Universidad Autónoma de Puebla Puebla, Mexico; ^8^Departamento de Biología Celular, Cinvestav Mexico City, Mexico

**Keywords:** motoneuron, GABA_A_ receptors, tonic inhibition, monosynaptic reflex

## Abstract

Motoneurons, the final common path of the Central Nervous System (CNS), are under a complex control of its excitability in order to precisely translate the interneuronal pattern of activity into skeletal muscle contraction and relaxation. To fulfill this relevant function, motoneurons are provided with a vast repertoire of receptors and channels, including the extrasynaptic GABA_A_ receptors which have been poorly investigated. Here, we confirmed that extrasynaptic α5 subunit-containing GABA_A_ receptors localize with choline acetyltransferase (ChAT) positive cells, suggesting that these receptors are expressed in turtle motoneurons as previously reported in rodents. In these cells, α_5_GABA_A_ receptors are activated by ambient GABA, producing a tonic shunt that reduces motoneurons’ membrane resistance and affects their action potential firing properties. In addition, α_5_GABA_A_ receptors shunted the synaptic excitatory inputs depressing the monosynaptic reflex (MSR) induced by activation of primary afferents. Therefore, our results suggest that α_5_GABA_A_ receptors may play a relevant physiological role in motor control.

## Introduction

Extrasynaptic GABA_A_ receptors play important roles in supra-spinal nuclei of the Central Nervous System (CNS) regulating neuron excitability and network activity by tonically inhibiting and shunting mature neuronal membranes, which set the threshold for action potential generation and temporal window for synaptic integration (Farrant and Nusser, 2005; Wlodarczyk et al., 2013). These receptors are conformed mainly by α_4_, α_5_ and α_6_ subunits, although sometimes they may contain α_2_ and α_3_ subunits (Farrant and Nusser, 2005). Though all these subunits are also expressed in the spinal cord their function is poorly understood (Persohn et al., 1991; Wisden et al., 1991; Ma et al., 1993; Ruano et al., 2000; Mody and Pearce, 2004; Petri et al., 2005; Delgado-Lezama et al., 2013; Andres et al., 2014; Loeza-Alcocer et al., 2014; Bravo-Hernández et al., 2016).

In a previous work, we investigated the role of high-affinity GABA_A_ receptors in motor behavior. We found that motoneurons presented a tonic current mediated by high affinity GABA_A_ receptors activated by ambient GABA (Castro et al., 2011a). Moreover, we demonstrated that blockade of furosemide-sensitive high affinity GABA_A_ receptors facilitated the monosynaptic reflex (MSR; Bautista et al., 2010). We have also provided evidence that motoneurons express a tonic current mediated by α_6_ subunit-containing GABA_A_ receptors (Andres et al., 2014). Likewise, several immunohistochemical and *in situ* hybridization studies have shown that α_5_ subunit-containing GABA_A_ (α_5_GABA_A_) receptors are expressed in motoneurons although its role is presently unknown (Persohn et al., 1991; Wisden et al., 1991; Ma et al., 1993; Ruano et al., 2000; Mody and Pearce, 2004; Petri et al., 2005; Loeza-Alcocer et al., 2014). Thus, we hypothesized that α_5_GABA_A_ receptors modulating the excitability of motoneurons play a very important role in motor control. In the present report, we investigate whether α_5_GABA_A_ receptors are expressed in motoneurons and mediate a GABAergic tonic current that control its excitability and modulate the MSR, using the turtle spinal cord as a model system. We found that α_5_GABA_A_ receptors are tonically active by ambient GABA and mediate a tonic current that reduce motoneurons’ excitability, decrease the input resistance and increase the rheobase. In addition, here we show that α_5_GABA_A_ receptors depress the MSR.

## Materials and Methods

### Preparation

Forty adult turtles (*Trachemys scripta spp*, 15–20 cm carapace length) were anesthetized with pentobarbital (100 mg/kg, i.p.). The plastron was opened and the blood removed by intraventricular perfusion with Ringer solution (~10°C) of the following composition (in mM): 120 NaCl, 5 KCl, 15 NaHCO_3_, 3 CaCl_2_, 2 MgCl_2_ and 20 glucose saturated with 2% CO_2_ and 98% O_2_ to attain a pH value of 7.6. The lumbar spinal enlargement was isolated by a laminectomy and cut transversally to obtain slices of 2–3 mm and 300 μm thick. For intracellular and patch-clamp recordings, the slices were placed in a chamber and superperfused with Ringer solution (20–22°C). For the extracellular recording two segments of the lumbar spinal cord in continuity with the dorsal and ventral roots were dissected out. At the end of the dissection, the animals were rapidly euthanized by decapitation.

The animals were provided by the National Mexican Turtle Center located in Mazunte, Oaxaca (Mexico) with the authorization DGVS-03821/0907 by the Federal Government Ministry of Environment and Natural Resources (Semarnat). In addition, this study was approved by the Institutional Animal Care and Use Committee (Cinvestav, Mexico City, Mexico; Protocol 0098-16) and followed the guidelines for ethical matters (Drummond, 2009).

### Electrophysiology

Motoneurons were recorded intracellularly in slices of 2–3 mm thick with a sharp electrode (20–40 MΩ) filled with potassium acetate (0.8 M) and KCl (0.2 M). Cells were classified as motoneurons if their input resistance was lower than 80 MΩ, presented an action potential waveform with a fast and slow posthyperpolarization and show adaptation during repetitive action potential firing (Hounsgaard et al., 1988; Delgado-Lezama et al., 2004). In order to evaluate the excitability of motoneurons, intracellular supra-threshold current pulses were applied in control Ringer and in presence of L-655,708, a selective α_5_GABA_A_ receptor inverse agonist (pKi 9.3; Quirk et al., 1996).

**Figure 1:**
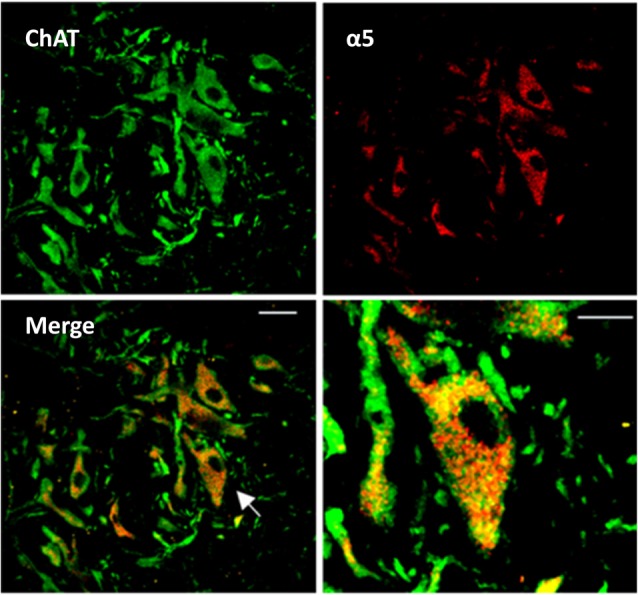
Expression of α_5_GABA_A_ receptors in spinal motoneurons. Representative confocal images from a slice of the adult turtle spinal cord. Neurons positive to choline acetyltransferace immunoreactivity are shown in green (ChAT, left upper panel), and α_5_GABA_A_ receptors in red (right upper panel). Superposition of the two images revealing the expression of α_5_GABA_A_ receptors on the soma of motoneurons is shown in the left lower panel (Merge). Right lower panel, digital amplification of a single neuron (arrow in left lower panel) shows the location of α_5_GABA_A_. Scale bar, 50 μm.

In other series of experiments, the presence of the GABAergic tonic current was determined in motoneurons by using the visualized patch clamp technique in its whole-cell configuration. The electrodes were made from thick-walled borosilicate glass capillaries using a Sutter programmable horizontal micropipette puller (Sutter Instrument). The patch pipettes with resistance of 5–10 MΩ were filled with the following solution (in mM): 122 CsCl; 5 Na_2_-ATP; 2.5 MgCl_2_; 0.0003 CaCl_2_; 5.6 Mg-gluconate; 5 K-HEPES; 5 HEPES. Visualizing the ventral horn with the Olympus microscope (BX51W1) motoneurons were identified by their location and size (Figure 1) and then electrophysiological recordings were performed by using the MultiClamp-700B amplifier (Molecular Devices). The maximal acceptable series resistance compensation was 20%. As described earlier, these neurons presented action potentials with fast and slow posthyperpolarization and adaptation in their firing pattern. Recorded signals were digitized at 20 KHz, filtered using a 8-pole Bessel (3 KHz) and stored in the hard disk of a computer for off-line analysis.

In additional experiments, the MSR was recorded from two spinal cord segments. The dorsal and ventral roots were suctioned by glass pipettes connected to a source of constant current and to a differential AC amplifier (Grass Instruments), respectively. The dorsal root (DR9) were stimulated with a rectangular current pulse (0.5 ms). The threshold was defined as the minimum stimulus intensity that elicits a measurable ventral root potential. Unless otherwise stated, the recordings shown were the average of 10 stimuli applied every 30 s. The MSR was recorded with a bandwidth of 0.1 Hz to 10 KHz, digitized at 10 KHz and stored for off-line analysis.

### Spinal Cord Immunostaining

Spinal cord sections of 30 μm were obtained as described previously (Castro et al., 2011b) and first incubated with an anti-choline acetyltransferase (anti-ChAT) primary antibody (24 h at 4°C, 1:50, Millipore) and then revealed using a FITC donkey anti-goat secondary antibody (2 h at room temperature, 1:200, Jackson ImmunoResearch). Subsequently, sections were incubated with an anti-α5 subunit GABA_A_ receptor primary antibody (2 h at 4°C, Sigma; 1:100 dilution), and then exposed 1 h to the secondary antibody (1:200; Dylight 549-conjugated anti-rabbit IgG; Jackson ImmunoResearch). Samples were examined using confocal laser scanning microscopy (Leica TCS SP2, Leyca Microsystems). Images were obtained using the 40× oil immersion plan apochromatic objective (NA 0.8) and a subsequent digital amplification using the ImageG software.

### Drugs

GABA_A_ receptors were activated with GABA (10–60 μM) and blocked with picrotoxin (100 μM) and L-655,708 (20 μM) applied to the bath solution. Ionotropic glutamatergic and glycinergic receptors were blocked with 6-cyano-7-nitroquinoxaline-2, 3-dione (CNQX; 20 μM) and (2R)-amino-5-phosphonovaleric acid (APV; 40 μM) and strychnine (2 μM), respectively. All drugs used in this study were purchased from Sigma-Aldrich.

### Analysis

The effect of GABA_A_ receptor activation or blockade in motoneurons recorded intracellularly was quantified by measuring the input resistance and excitability in the absence and presence of L-655,708. The input resistance was determined as the slope of the fitted line to the *I-V* plot. The excitability was evaluated by plotting the current intensity vs. the number of action potentials produced by supra-threshold intracellular current pulses. A change in excitability was indicated by a horizontal shift of the resulting curve. To determine the effect of L-655,708 on the MSR and the dorsolateral funiculus (DLF) induced EPSP we measured the EPSP amplitude and the area under the curve of the MSR in control Ringer and in presence of the drug. The average amplitude of 30 EPSPs and the MSR area were calculated for each condition. Differences between means were determined by the unpaired Student’s *t*-test. Means were considered statistically different when *p* < 0.05.

The mean holding current recorded in voltage clamp experiments was calculated by generating all-point histograms of the current values recorded for 5 s in control Ringer and in the presence of L-655,708. A Gaussian distribution was fitted to the histograms. Changes in the holding current were determined as the difference between the means of the Gaussians fitted to the histograms. Differences between Gaussian means were determined by the Student’s *t*-test. Means were considered statistically different when *p* < 0.05. Values are presented as the mean ± SEM.

Intracellular recordings were performed in 2–3 mm thick slices while the patch clamp recordings were made in slices 300 μm thick. The results are not comparable because the dendritic tree of motoneurons is severed in thinner slices. In addition, they are recorded at a maximum depth of 40 μm, therefore it is not easy to evoke dendritic EPSPs by DR9 stimulation. By the contrary, intracellular recordings of motoneurons were made in normal Ringer at a depth of 200–300 μm in which the dendritic tree is almost intact. Likewise, the extracellular GABA concentration is enough to activate the high affinity GABA_A_ receptors as was evidenced by the decrease in the rehobase and increase in input resistance and excitability, reflected by a leftward shift in the excitability curve when L-655,708 was added. Therefore both set of data were analyzed independently.

## Results

### α_**5**_GABA_**A**_ Receptor Expression in Turtle Spinal Motoneurons

Our first approach to evaluate the activity of α_5_GABA_A_ receptors in motoneurons was to determine their cellular expression. It is worth mentioning that the presence of α_5_GABA_A_ receptors in mammalian motoneurons has been suggested previously (Persohn et al., 1991; Wisden et al., 1991; Ruano et al., 2000). In order to determine whether the α5 subunit is also expressed specifically in turtle motoneurons as previously reported in rodents, immunohistochemical staining was performed on transverse slices of the turtle lumbar spinal cord. The results of this analysis showed that the α_5_GABA_A_ immunostaining is prominent in cells expressing ChAT (a marker for motoneurons), where signal was dispersedly distributed in the soma, sparing the nucleus (Figure 1). Therefore, our results suggest that extrasynaptic α_5_ subunit-containing GABA_A_ receptors are also expressed in choline acetyltransferase (ChAT) turtle positive cells as in rodents. No labeling was seen in absence of the primary antibody or in presence of its corresponding antigenic peptide (data not shown).

### α_5_GABA_A_ Receptors Mediate a Tonic Inhibitory Current in Motoneurons

**Figure 2:**
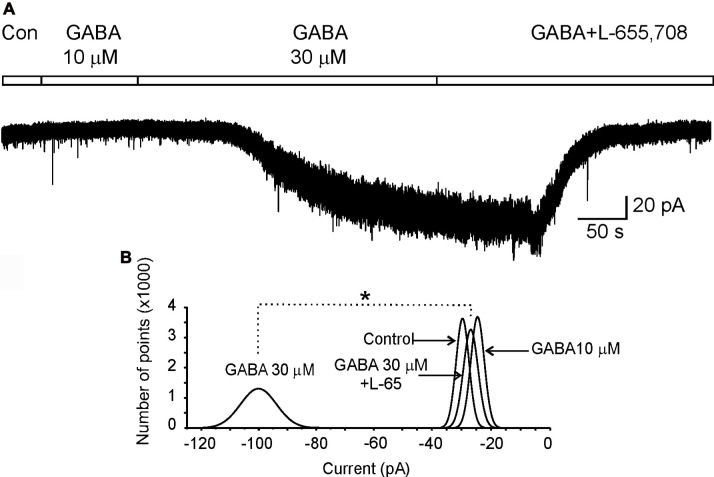
α_5_GABA_A_ receptors mediate a tonic inhibitory current in motoneurons.** (A)** Holding current recorded in a motoneuron clamped at −70 mV in control (Con) Ringer or the presence of GABA at 10 μM and 30 μM plus L-655,708. **(B)** Gaussian curves fitted to the all-points histograms of current values obtained from the holding current recorded in control Ringer and in the presence of GABA (10 μM and 30 μM) plus L-655,708 (20 μM) as indicated. Asterisk indicates a statistical difference between means of holding currents recorded with GABA 30 μM and L-655,708 (*p* < 0.05, unpaired Student’s *t*-test).

Next, we decided to perform electrophysiological recordings to characterize the possible role of the α_5_GABA_A_ receptors in spinal cord motoneurons. To facilitate the characterization of the α_5_GABA_A_ receptors, the tonic current was enhanced by application of GABA (30 μM) to the perfusion solution. This was based on the demonstration that the receptors display similar sensitivity and kinetics when exposed to either endogenous or exogenous GABA application (Bai et al., 2001). As can be seen in Figure 2, whole-cell patch clamp recordings with a high Cl^−^ internal solution showed a GABA-activated inward current that did not display inactivation.

Previous studies have shown that α_5_GABA_A_ receptors mediate a tonic current in different nuclei of the CNS (Farrant and Nusser, 2005; Bonin et al., 2007). To confirm that these receptors also mediate a tonic current in motoneurons, cells were recorded in presence of GABA (30 μM) in combination with a cocktail containing (in μM): 2 strychnine, 20 CNQX and 20 APV, to block the activity of glycine, AMPA-kainate and NMDA receptors, respectively. In these conditions, we showed that the holding current of a motoneuron, maintained at a holding potential (*V*_h_) of −70 mV and recorded for 50 s in control conditions, was not changed in the presence of GABA (10 μM; Figure 2). Nevertheless, when GABA concentration was raised to 30 μM, after about 100 s an inward current of 75 ± 0.06 pA was activated, which was associated to an increase in noise possibly due to stochastic activation of GABA_A_ receptors (Brickley et al., 2001). Interestingly, after adding the α_5_GABA_A_ inverse agonist L-655,708 the holding current returned to the control level in amplitude and noise as can be seen in the normal fitted current histograms (Figure 2). A similar result was observed in a total of four motoneurons. We found that L-655,708 did not block all the current activated by GABA in two neurons; and the remaining current was blocked by picrotoxin (100 μM). These results suggest that the tonic current evoked in motoneurons might be mediated by more than one type of α subunit. As we previously showed, this subunit could be α_6_ (Andres et al., 2014).

### α_5_GABA_A_ Receptors Modulate Motoneuron Excitability

It has been shown that tonic GABAergic current modulates neuronal excitability by shunting the membrane and decreasing the membrane time constant (Mitchell and Silver, 2003; Wlodarczyk et al., 2013). Therefore, we next sought to determine whether the α_5_GABA_A_ receptors were performing a similar role, by evaluating the action of L-655,708 (20 μM) on the passive and active properties of these neurons recorded intracellularly. The cells selected for this series of experiments presented an input resistance of 21 ± 3.6 MΩ, an action potential waveform with the typical fast and slow post-hyperpolarization, and also showed adaptation of the firing pattern produced by a long intracellular depolarizing current pulse, as occurs in motoneurons according to previous reports (Hounsgaard et al., 1988; Delgado-Lezama et al., 2004).

**Figure 3:**
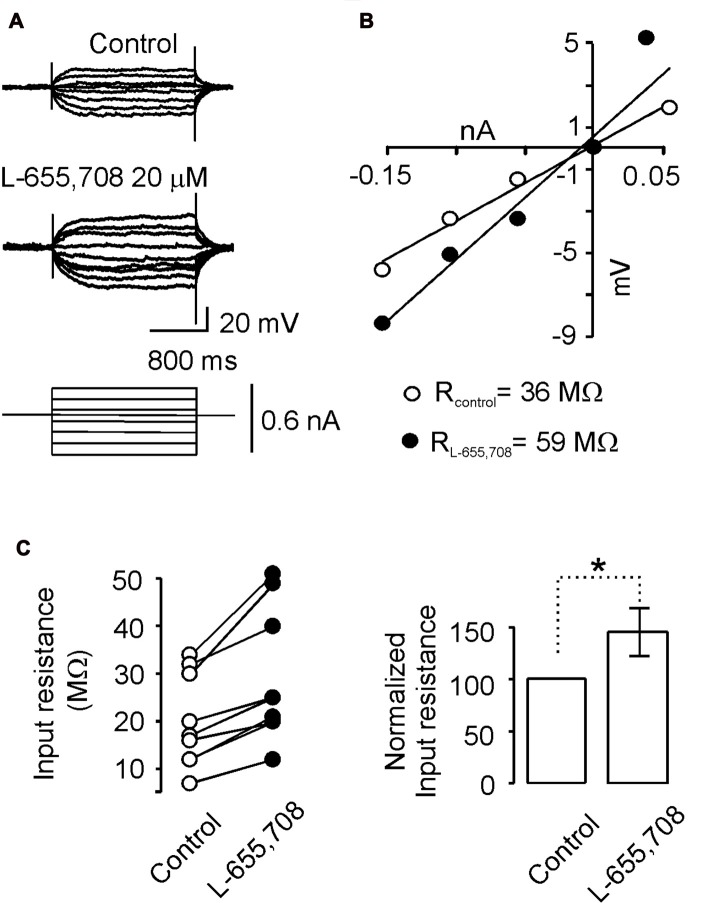
α_5_GABA_A_ receptors modulate passive membrane properties of motoneurons. **(A)** Current-voltage responses recorded intracellularly from motoneurons in Ringer solution (control) and in the presence of L-655,708 (20 μM). **(B)** Input resistance was estimated via linear regression applied to the I-V plot data. **(C)** Left panel, input resistance values measured in control Ringer and in presence of L-655,708 are shown. Right panel, bar graph showing the mean input resistance of nine motoneurons in the absence and presence of L-655,708. *Indicates that the two groups of data are statistically different (*p* < 0.05, unpaired Student’s *t*-test).

A typical response of one motoneuron is presented in Figure 3A, which shows that the voltage response to the same current pulses was more intense in the presence of L-655,708 to that recorded in control Ringer. This action of L-655,708 was evaluated in the *I-V* plot. Regression analysis of the individual slopes of the data showed that the input resistance increased from 36 MΩ to 59 MΩ after blockade of α_5_GABA_A_ receptors (Figure 3B). Similar responses were recorded in a total of nine motoneurons. On average, the input resistance increased in 32 ± 7% in the presence of L-655,708 compared to the control (Figure 3C; *n* = 9; *p* < 0.05, unpaired Student’s *t*-test). These results suggest that α_5_GABA_A_ receptors tonically activated by ambient GABA are shunting the membrane of motoneurons.

**Figure 4:**
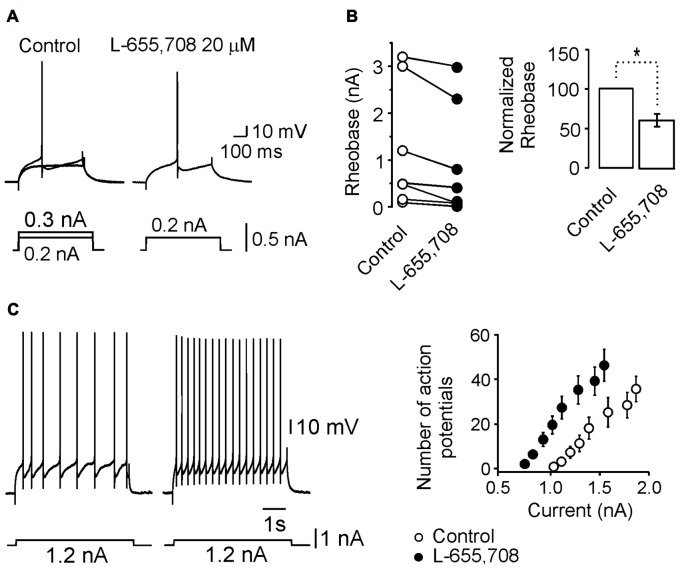
L-655,708-sensitive GABA_A_ receptors modulate motoneuron excitability. **(A)** Minimum current (rheobase) required to evoke an action potential under both control and L-655,708 conditions. **(B)** Comparison of the mean rheobase values of seven motoneurons in both experimental conditions. **(C)** Left panel, action potential firing in response to the same current pulse under control conditions and in the presence of the L-655,708. Right panel, depolarizing current pulses vs. number of action potentials evoked in seven motoneurons in control Ringer (empty circles) and in the presence of L-655,708 (solid circles). *Indicates that the two groups of data are statistically different (*p* < 0.05, unpaired Student’s *t*-test).

We next wanted to know whether the change in input resistance generated by the α_5_GABA_A_ receptor activity was sufficient to modify the action potentials firing. As can be seen in Figures 4A,B, the minimum current injected to generate an action potential (rheobase) decreased in 22 ± 5.3% with respect to control (*n* = 7; *p* < 0.05, unpaired Student’s *t*-test) in presence of L-655,708. As a consequence, in presence of the drug, the number of action potentials activated in response to depolarizing current of increasing intensities was augmented (Figure 4C). The action of L-655,708 on the firing properties of motoneurons may be better visualized in the right panel of Figure 4C where blockade of α_5_GABA_A_ receptors caused a leftward shift in the excitability curve. The number of action potentials was statistically different for every current in both conditions (*n* = 7; *p* < 0.05, unpaired Student’s *t*-test). These results suggest that α_5_GABA_A_ receptors are tonically modulating the firing properties of spinal motoneurons.

### Postsynaptic α_5_GABA_A_ Receptors Modulate Synaptic Transmission

**Figure 5:**
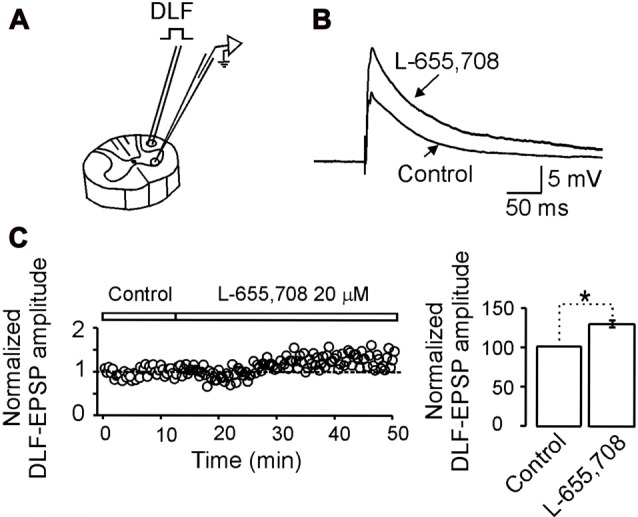
α_5_GABA_A_ receptors control the excitatory synaptic strength between dorsolateral funiculus (DLF) and motoneurons.** (A)** Scheme illustrating the site where the DLF was electrically stimulated to evoke excitatory postsynaptic potentials (EPSPs) in a motoneuron recorded intracellularly in the ventral horn of a spinal cord slice. **(B)** EPSPs recorded from a motoneuron in control Ringer and in the presence of L-655,708. **(C)** Left panel, time course of the normalized EPSP amplitude recorded in control Ringer and in the presence of L-655,708. Right panel, bar graph shows the percentage increase of the EPSP amplitude in presence of the drug recorded in 10 motoneurons. Asterisk indicates statistical difference between means of EPSP amplitude in control Ringer and L-655,708 (*p* < 0.05, unpaired Student’s *t*-test).

In a previous study, we showed that the functional role of the tonic shunting produced by high affinity GABA_A_ receptors might be associated to a depression of excitatory synaptic potentials at the dendrites (Delgado-Lezama et al., 2004). To investigate whether the α_5_GABA_A_ receptors are participating in this type of synaptic modulation, we next decided to investigate the action of L-655,708 on the excitatory postsynaptic potential (EPSP) evoked by electrical stimulation of the DLF, which do not express GABA_A_ receptors (Delgado-Lezama et al., 2004). Figure 5A illustrates the site where the DLF was electrically stimulated to evoke the EPSPs in a motoneuron recorded intracellularly in the ventral horn of a spinal cord slice. Figure 5B shows the EPSPs recorded from a motoneuron in control Ringer and in the presence of L-655,708. On average, the EPSP amplitude was facilitated by 19 ± 2.5% with respect to control (Figure 5C; *n* = 10; *p* < 0.05, unpaired Student’s *t*-test). These results suggest that the tonic activity of α_5_GABA_A_ receptors modulate the excitatory synaptic potential of motoneurons.

### α_5_GABA_A_ Receptors Modulate the Monosynaptic Reflex (MSR)

**Figure 6:**
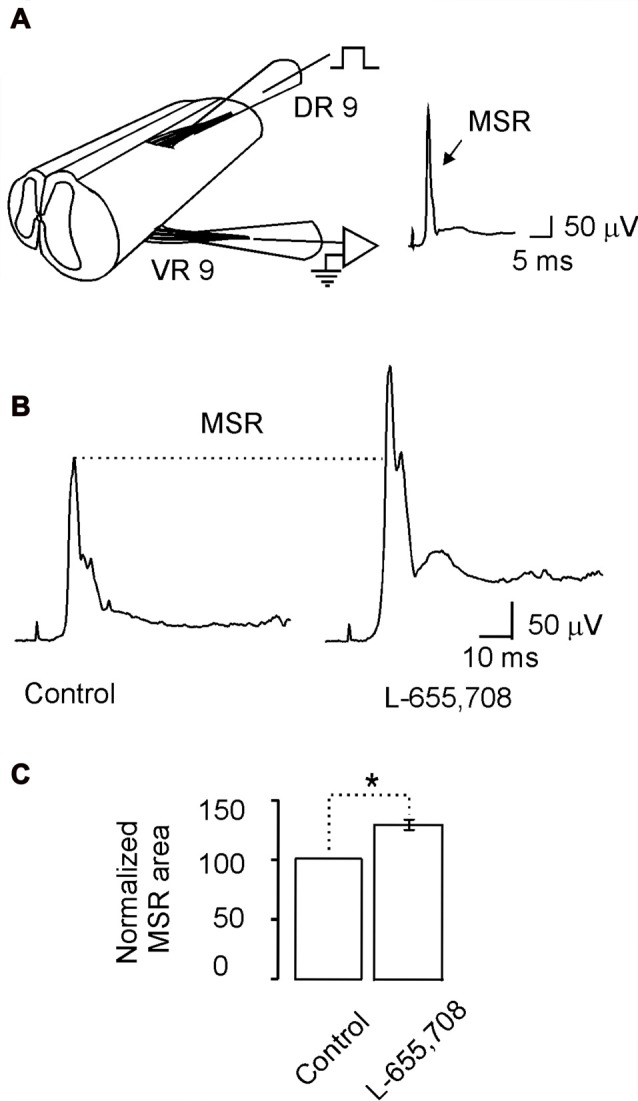
The monosynaptic reflex (MSR) is modulated by L-655,708-sensitive GABA_A_ receptors. **(A)** Scheme showing the spinal cord in continuity with the dorsal and ventral roots to stimulate electrically the primary afferents and record the MSR, respectively. **(B)** The MSR recorded in control Ringer and in the presence of L-655,708. **(C)** Bar plot shows the normalized MSR area evaluated in nine motoneurons. Asterisk indicates statistical difference between means of the MSR area in control Ringer and L-655,708 (*p* < 0.5, Student’s *t*-test).

Previously we also showed that high affinity GABA_A_ receptors may modulate the MSR (Bautista et al., 2010). Therefore, if α_5_GABA_A_ receptors are regulating the motoneuron excitability we wonder whether they can also modulate the MSR. Figure 6A shows the experimental preparation for MSR recording. Stimulation of the DR9 evoked a ventral potential (VR9) consisting of mono- and poly-synaptic reflexes (Figure 6B). Interestingly, both responses were facilitated after the application of L-655,708 (Figure 6B). On average, the MSR was facilitated in about 20 ± 4% compared with the control (Figure 6C; *n* = 9; *p* < 0.05, unpaired Student’s *t*-test). These results suggest that α_5_GABA_A_ receptors tonically activated by ambient GABA play an important role in motor control.

## Discussion

In the present report we show that the α_5_GABA_A_ receptors tonically activated by ambient GABA produce a tonic inhibitory current that modulates the passive and active properties of the turtle spinal motoneurons. These data suggest that GABA_A_ receptors may play an important role in motor control.

### α_5_GABA_A_ Receptors Mediate Tonic Inhibitory Currents in Motoneurons

In order to characterize the tonic current, we perfused exogenously GABA (30 μM) into the experimental preparation. Interestingly, the current activated in this condition showed an increase in noise with respect to the control current which returned to the basal level after the blockade of the α_5_GABA_A_ receptors with L-655,708. This finding agree with previous reports in the hippocampus, the dorsal horn and the intermediate area of the spinal cord were tonic inhibitory currents are mediated meanly by α_5_GABA_A_ receptors (Takahashi et al., 2006; Bonin et al., 2007; Glykys and Mody, 2007; Wang et al., 2008; Castro et al., 2011b; Perez-Sanchez et al., 2016). Interestingly, in two neurons the tonic current was resistant to L-655,708, though it was sensitive to blockade by picrotoxin (100 μM). Previously, we had observed a similar result by applying furosemide to block α_6_GABA_A_ receptors in motoneurons. In this case, the furosemide-resistant tonic current was also completely blocked by 100 μM picrotoxin application (Andres et al., 2014). Taken together, these data suggest that more than one extrasynaptic GABA_A_ receptor might be mediating the tonic current in motoneurons as observed in pyramidal cells of the hippocampus (Mody and Pearce, 2004; Farrant and Nusser, 2005). It is therefore reasonable to propose that extrasynaptic α_5_GABA_A_ and α_6_GABA_A_ receptors are expressed in motoneurons and are tonically activated by ambient GABA.

### α_5_GABA_A_ Receptors Modulate Passive and Active Properties of Motoneurons

Blockade of the α_5_GABA_A_ receptors by L-655,708 increased the input resistance of motoneurons which implies a α_5_GABA_A_-shunting tonic inhibition. When this inhibition is removed by L-655,708 action, important effects on the motoneuron excitability are unmasked. For instance, the rheobase significantly decreases and the number of action potentials is increased (Figure 4). This observation is in agreement with the result observed in the hippocampus where α_5_GABA_A_ receptors regulate the intrinsic excitability of pyramidal cells (Bonin et al., 2007), and in cerebellar granule cells where a tonic inhibitory current, recorded in a free moving mouse, controls its excitability lowering the excitatory synaptic potentials and producing a leftward shift on the frequency–current relationship (Chadderton et al., 2004).

It is possible that shunting inhibition may not be involved in the neuronal gain, but GABAergic tonic shunt might reduce neuronal activity in response to excitatory synaptic input. Indeed, L-655,708 increased the amplitude of EPSPs evoked by electrical stimulation of the DLF (Figure 5). In this context, our interpretation is that tonic activity of α_5_GABA_A_ receptors might be a mechanism in the motoneuron to control synaptic input by altering its membrane time constant, thereby narrowing the time window to integrate excitatory synaptic inputs, serving as a filter to preserve the accuracy of the motoneuron response.

### The Monosynaptic Reflex Is Modulated by α_5_GABA_A_ Receptors

We found also that the α_5_GABA_A_ inverse agonist L-655,708 facilitates the MSR. Interestingly, it has been reported that furosemide facilitates the MSR by blocking α_6_GABA_A_ receptors, although simultaneous electrophysiological recordings showed that the dorsal root potential (DRP) was not affected (Bautista et al., 2010). In contrast, L-655,708 depressed the DRP, although by using the excitability test we found that primary afferent depolarization associated with presynaptic inhibition was not affected by the drug (Loeza-Alcocer et al., 2013). This means that the MSR facilitation was not due to blockade of presynaptic inhibition mediated by synaptic GABA_A_ receptors. Therefore, having shown that α_5_GABA_A_ receptors presumably control motoneuron excitability in a similar fashion than α_6_GABA_A_ receptors, it is therefore reasonable to propose that these receptors may be also modulating the MSR.

### Functional Implications

It has been reported that extrasynaptic high affinity GABA_A_ receptors expressed in motoneurons may play an important role in motor control by imposing a tonic shunting that decreases excitability and prevent anomalous firing of action potentials (Bautista et al., 2010). This function was evidenced by studying the regulation of the MSR which is useful to determine motoneuron excitability (Rekling et al., 2000). Interestingly, α_5_GABA_A_ as well as α_6_GABA_A_ receptors tonically activated by ambient GABA decrease motoneurons excitability by shunting the membrane which prevents its anomalous activation. This is suggested by the facilitation of the MSR followed by a long lasting activation of motoneurons after blockade of these receptors (Bautista et al., 2010).

Taking into consideration that α_5_GABA_A_ receptors are also expressed in premotor interneurons (Castro et al., 2011a), our results suggest that these receptors might play an important role in regulating the neuronal network involved in motor control.

## Author Contributions

MC-B, EL-A, CAC, RF and RD-L: conceived and designed the experiments. MC-B, EL-A, CAC and PO: performed the experiments. MC-B, EL-A, CAC, PO and RDL: analyzed the data. MC-B, EL-A, CAC, DE-V, VG-S, EM, RF and RD-L: contributed reagents/materials/analysis tools. MC-B, EL-A, CAC, PO, EM, VG-S, RF and RD-L: contributed to the writing of the manuscript.

## Conflict of Interest Statement

The authors declare that the research was conducted in the absence of any commercial or financial relationships that could be construed as a potential conflict of interest.
